# Supplementary material for: Conditional chemoconnectomics (cCCTomics) as a strategy for efficient and conditional targeting of chemical transmission
Source: eLife. 2024 Apr 30;12:RP91927. doi: 10.7554/eLife.91927 (PMC11060718; doi:10.7554/eLife.91927)
Supplement: Figure 3—source data 3. [file elife-91927-fig3-data3.zip › Figure 3 source data3 part 2.zip!/Figure 3-Source data 3-part2/1060-9-CRISPResso_Report_q0_g4o36.zip!/CRISPResso_on_1060_12/3b.Insertion_deletion_substitutions_size_hist.pdf]

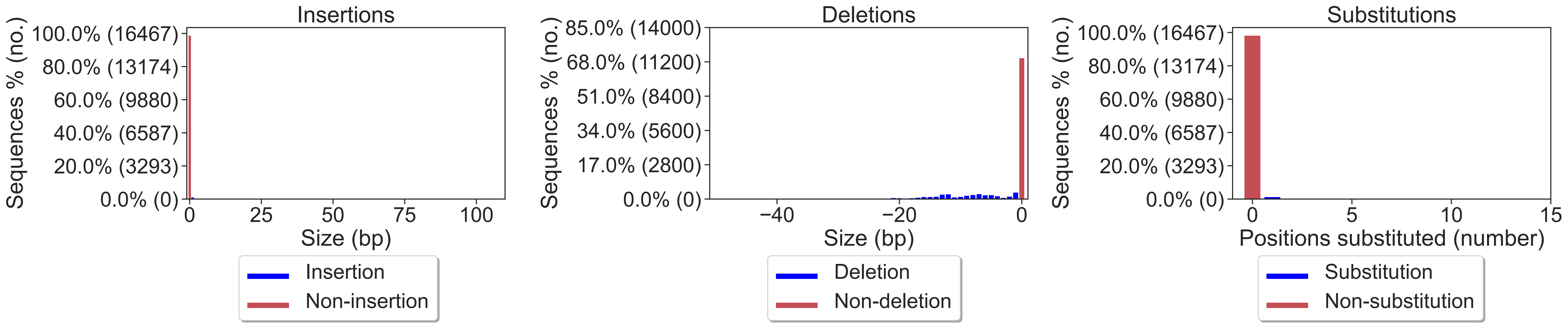
Insertions
Sequences % (no.)
100.0% (16467)
80.0% (13174)
60.0% (9880)
40.0% (6587)
20.0% (3293)
0.0% (0)
0
25
50
75
100
Size (bp)
Insertion
Non-insertion
Deletions
Sequences % (no.)
85.0% (14000)
68.0% (11200)
51.0% (8400)
34.0% (5600)
17.0% (2800)
0.0% (0)
−40
−20
0
Size (bp)
Deletion
Non-deletion
Substitutions
Sequences % (no.)
100.0% (16467)
80.0% (13174)
60.0% (9880)
40.0% (6587)
20.0% (3293)
0.0% (0)
0
5
10
15
Positions substituted (number)
Substitution
Non-substitution
